# An Integrated Two-Stage Strategy for Efficient Lactic Acid Production via Ionic Liquid-Assisted Mechanochemical Pretreatment and Catalytic Conversion

**DOI:** 10.3390/molecules31162922

**Published:** 2026-08-21

**Authors:** Zheyuan Zhang, Chuan Li Lee, Jianrong Shan, Haixin Guo

**Affiliations:** 1 College of Resource and Environment, Hubei University, 368 Youyi Avenue, Wuchang District, Wuhan 430062, China; 2 Agro-Environmental Protection Institute, Ministry of Agriculture and Rural Affairs, No. 31 Fukang Road, Nankai District, Tianjin 300191, China; 82101221187@caas.cn; 3 Faculty of Biotechnology and Biomolecular Sciences, Universiti Putra Malaysia, Serdang 43400, Selangor, Malaysia

**Keywords:** ball-milling, cellulose, lactic acid, ionic liquid, rehydrated hydrotalcite

## Abstract

Efficient conversion of cellulose to value-added lactic acid remains challenging because the rigid crystalline structure of cellulose limits its accessibility and subsequent conversion. This study developed an integrated strategy combining ionic liquid-assisted ball milling with catalytic lactic acid production. Ionic liquid type, ball-milling time, ionic liquid concentration, and glucose addition were examined during cellulose pretreatment. Among the ionic liquids tested, 1-butyl-3-methylimidazolium acetate ([Bmim][OAc]) provided the highest apparent water-soluble fraction. After 60 min of ball milling with 0.17 M [Bmim][OAc], the apparent water-soluble fraction increased from 5.1% for untreated cellulose to 63.7%, while the crystallinity index decreased from 78.1% to 44.0%. The catalytic stage was then evaluated using D-glucose as a model substrate with rehydrated hydrotalcite (r-HT) in a Ba(OH)_2_ medium. The r-HT/Ba(OH)_2_ system achieved the highest lactic acid yield of 43.6 C-% at 100 °C for 2 h, compared with 9.1 C-% for r-HT and 38.2 C-% for Ba(OH)_2_ alone. These results demonstrate the potential of this integrated strategy to improve cellulose accessibility while enhancing lactic acid formation from glucose, providing a basis for further development of cellulose-to-lactic-acid conversion pathways.

## 1. Introduction

The progressive depletion of fossil fuel reserves and the increasing concern over environmental issues have encouraged research efforts toward sustainable technologies for renewable feedstock conversion [1,2,3]. Cellulose, one of the most abundant renewable organic carbon sources on Earth, with an estimated annual production exceeding 50 billion tons, represents a promising non-food-competing feedstock for the production of high-value chemicals [4,5]. However, its extensive inter-chain hydrogen bonding networks and highly ordered crystalline structure restrict reagent accessibility and hinder chemical conversion [1]. Therefore, effective pretreatment methodologies are required to modify the cellulose structure and improve its accessibility for subsequent chemical conversion [6].

Lactic acid (LA) is a vital platform chemical with widespread applications in the food, pharmaceutical, cosmetic, and biodegradable polymer industries [7]. The global lactic acid (LA) market is expected to expand substantially, with its market value projected to reach approximately USD 6.9 billion by 2030 [8]. At present, industrial LA production relies mainly on microbial fermentation of edible sugars, which can involve competition with food resources, relatively high operating costs, and limited productivity [9]. These limitations have encouraged interest in catalytic approaches for LA production from carbohydrate feedstocks, with recent studies highlighting the potential of heterogeneous solid catalysts [10]. In this context, cellulose-derived sugars provide an alternative feedstock for exploring catalytic LA production from non-edible biomass.

Mechanochemical pretreatment via ball milling has emerged as an effective approach for modifying the crystalline structure of cellulose. The mechanical energy generated through impact, compression, shear, and friction can reduce particle size and alter the hydrogen-bonding environment, thereby increasing cellulose accessibility [11,12]. Ball milling in the presence of acid or alkaline agents has also been reported to enhance cellulose solubility by promoting structural disruption and improving cellulose accessibility [13,14]. Furthermore, ionic liquids (ILs), particularly imidazolium-based ILs such as [Bmim][OAc], can interact with cellulose through their hydrogen-bonding properties, facilitating changes in the inter-chain hydrogen-bonding environment [15,16,17,18]. The basicity and hydrogen-bond properties of ILs are also important factors governing their interactions with substrates [19].

For the conversion of cellulose-derived sugars to LA, various catalytic approaches have been investigated. Lewis acids and transition-metal salts have been shown to promote isomerization and retro-aldol reactions, although the resulting yields are modest under relatively severe reaction conditions [20]. In contrast, homogeneous bases such as NaOH and Ba(OH)_2_, as well as hydrothermal approaches, demonstrate high activity through base-mediated isomerization and retro-aldol fragmentation [21]. Ba(OH)_2_ has also been reported to promote LA formation from sugars through base-mediated reaction pathways [7]. The synthesis of LA from dihydroxyacetone, a key intermediate, using alkaline-earth metal hydroxides further highlights the role of divalent cations in this reaction pathway [22]. From a practical perspective, solid-base catalysts such as calcined and rehydrated hydrotalcites (HTs) have attracted interest as heterogeneous catalysts for sugar conversion [23,24]. HT-derived materials provide tunable basic sites for sugar isomerization [25], while structure–performance studies of Mg–Al hydrotalcites have highlighted the influence of interlayer anion composition on catalytic activity [26]. These characteristics indicate the potential of rehydrated HT as a solid-base catalyst for glucose conversion to LA in the presence of Ba(OH)_2_.

However, the respective contributions of mechanical activation, IL–cellulose interactions, and glucose-assisted structural modification to cellulose accessibility and its apparent water-soluble fraction have yet to be fully established. In the catalytic stage, the performance of hydrotalcite-based solid bases for glucose conversion to LA in the presence of Ba(OH)_2_ also warrants further evaluation. Accordingly, the present study investigates ionic liquid-assisted ball milling for cellulose pretreatment and evaluates glucose conversion to LA using rehydrated hydrotalcite (r-HT)-based catalysts. The objectives were to (1) evaluate the effects of different ILs and glucose addition on the apparent water-soluble fraction of ball-milled cellulose; (2) examine the effects of ball-milling time and IL concentration together with the associated structural changes in cellulose; (3) examine the structural evolution of hydrotalcite during calcination and rehydration; and (4) evaluate the catalytic performance of HT-based catalysts for D-glucose conversion to LA and examine the associated reaction pathway.

## 2. Results and Discussion

### 2.1. Pretreatment of Cellulose with Different Ionic Liquids

**Figure 1:**
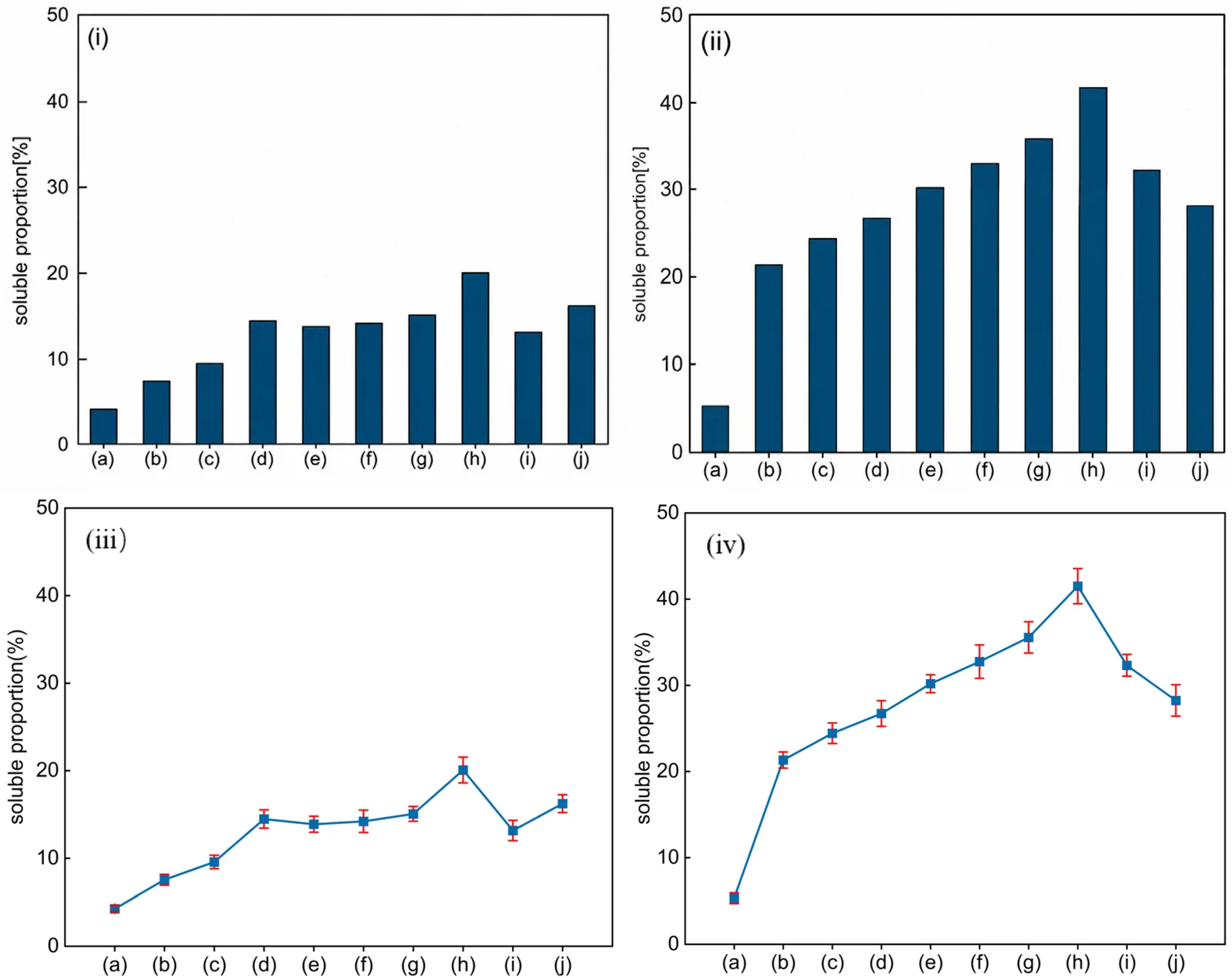
Soluble fraction of cellulose: (i) without glucose addition, (ii) with glucose addition, (iii) error bars for cellulose without glucose addition, and (iv) error bars for cellulose with glucose addition. Pretreatment conditions: (a) no milling, (b) no solvent, (c) sulfuric acid, (d) [Bmim]Cl, (e) [Bmim]OH, (f) [Bmim][HSO_4_], (g) [Bmim]Br, (h) [Bmim][OAc], (i) NaOH + [Bmim]Cl, and (j) H_2_SO_4_ + [Bmim]Cl. (Cellulose: 5 g; aqueous solution: 0.13 M, 10 mL; ball milling: 20 min for conditions (b)–(j)).

The soluble fractions of cellulose following pretreatment with different solvents are presented in Figure 1(i). In the absence of ball milling, the soluble fraction of raw cellulose was only 5.1%. After 20 min of ball milling without solvent addition, the soluble fraction increased to approximately 10%, suggesting that mechanical milling altered the cellulose structure and increased its accessibility to water. When various aqueous solutions were employed as impregnation solvents under identical 20-min ball milling conditions, ionic liquid solutions generally exhibited higher soluble fractions than pure water and sulfuric acid. Among the ionic liquids evaluated, [Bmim][OAc] achieved the highest soluble fraction of 20.1%, followed by [Bmim]Br and [Bmim]Cl.

As shown in Figure 1(i), the highest soluble fraction was obtained with [Bmim][OAc] (20.1%), followed by [Bmim]Br. The higher solubilization achieved with [Bmim][OAc] can be attributed to the high hydrogen-bond basicity of the acetate anion. Hydrogen-bond basicity has been identified as an important factor governing cellulose dissolution in ionic-liquid systems [19]. In particular, acetate anions can form strong hydrogen bonds with the hydroxyl groups of cellulose, which can weaken the hydrogen-bonding interactions within the cellulose structure and facilitate solubilization [27]. The lower soluble fraction obtained with [Bmim]Br compared with [Bmim][OAc] therefore indicates the stronger solubilizing effect of the acetate-based ionic liquid under the present pretreatment conditions. As shown in Figure 1(i), although acid- or base-assisted hydrolysis could potentially promote glycosidic-bond cleavage, no corresponding increase in the soluble fraction was observed when H_2_SO_4_ or NaOH was added to [Bmim]Cl under the conditions investigated. This finding suggests that the hydrogen-bond-disrupting capability of the ionic liquid anion plays a more important role than the additional acidic or basic hydrolysis under the pretreatment conditions investigated.

### 2.2. Effect of Glucose Addition on Cellulose Pretreatment

The soluble fractions of cellulose with added glucose following pretreatment are presented in Figure 1(ii). All samples with glucose addition exhibited higher soluble fractions than the corresponding samples without glucose addition (Figure 1(i)). Under identical pretreatment conditions, [Bmim][OAc] gave the highest soluble fraction of 41.5%, approximately twice that obtained without glucose addition (20.1%). The increase in soluble fraction indicates that glucose addition further affected the cellulose structure during ball milling. Previous work has shown that mechanochemical treatment can disrupt the hydrogen-bonding network within crystalline cellulose and promote the formation of water-soluble products [28]. The higher soluble fraction observed with glucose addition may therefore reflect further modification of the cellulose structure and increased formation of water-accessible soluble species during pretreatment.

**Figure 2:**
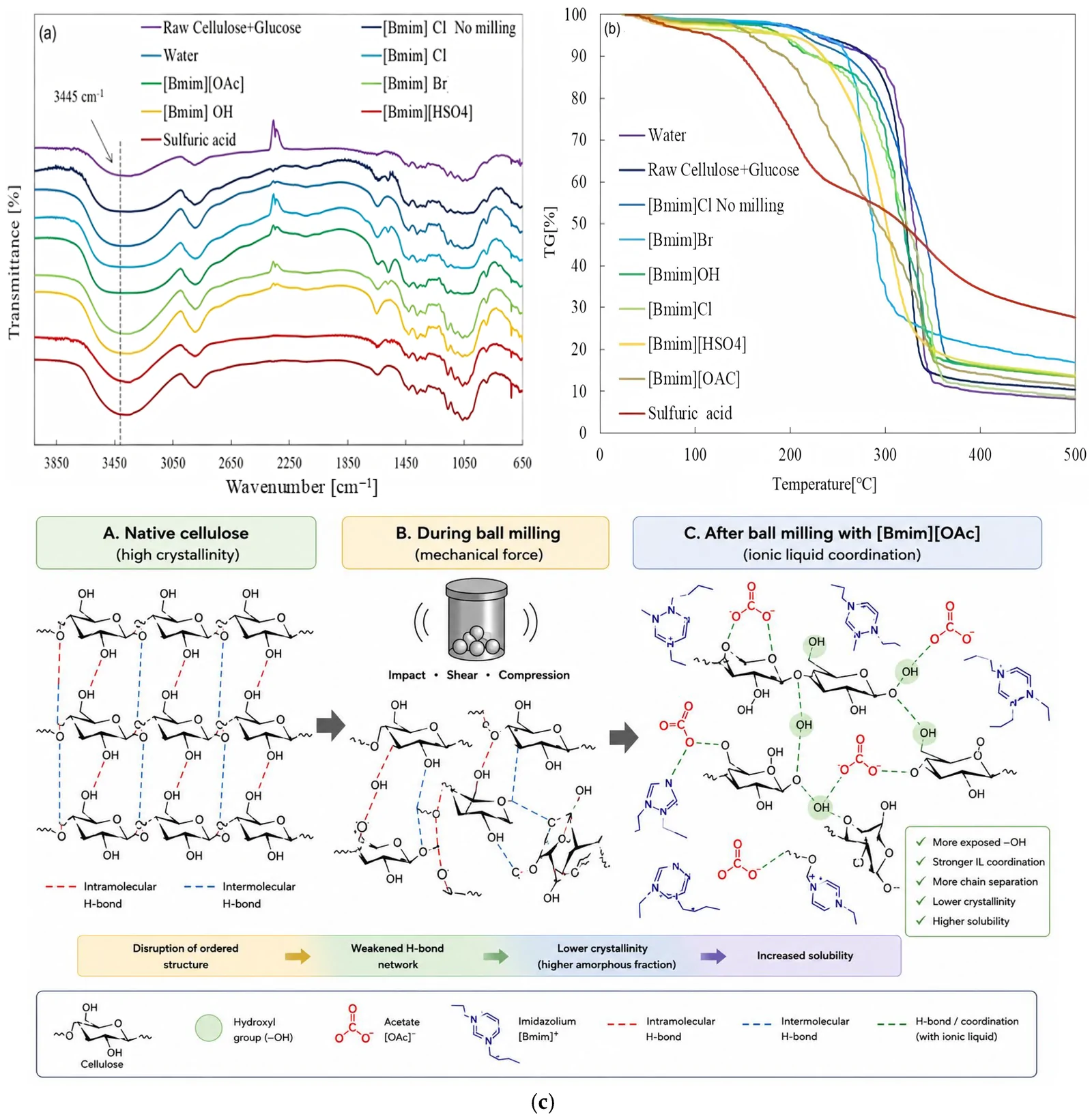
(**a**) FT-IR spectra of pretreated cellulose with glucose addition and (**b**) TG curves of pretreated cellulose with glucose addition (**c**) Schematic illustration of cellulose hydrogen bonding and its interaction with [Bmim][OAc] during ball milling. (Cellulose: 4.375 g, glucose: 0.625 g, aqueous solution: 0.13 M, 10 mL, ball milling: 20 min).

FT-IR analysis (Figure 2a) further revealed changes in the chemical environment of cellulose following pretreatment. Compared with the Raw Cellulose + Glucose sample, the pretreated samples showed changes in the broad O–H stretching region around 3445 cm^−1^, reflecting changes in the hydrogen-bonding environment of cellulose. Such changes in the O–H stretching band have been associated with modifications in the crystalline structure of cellulose [29]. These changes in the hydrogen-bonding environment may help explain the greater water accessibility within the cellulose structure and the increased soluble fractions observed after pretreatment, as shown in Figure 1(i).

TG analysis (Figure 2b) showed that most of the pretreated samples underwent their main thermal degradation at approximately 250–300 °C. In contrast, the sulfuric acid-treated sample (red line) exhibited degradation at a considerably lower temperature, indicating substantially lower thermal stability. This behavior may be associated with the strong-acid-induced decrystallization and depolymerization of crystalline cellulose, as previously reported [30]. Apart from that, the [Bmim][OAc]-treated sample also exhibited relatively lower thermal stability than several of the other ionic-liquid-treated samples. This behavior may be related to the ability of acetate anions to disrupt cellulose hydrogen bonds by forming new interactions with cellulose hydroxyl groups [31]. This interpretation is supported by the changes observed in the O–H stretching region in the FT-IR spectra (Figure 2a) and the higher soluble fraction obtained for [Bmim][OAc] (Figure 1(ii)).

### 2.3. Effect of Pretreatment Temperature and Time

**Figure 3:**
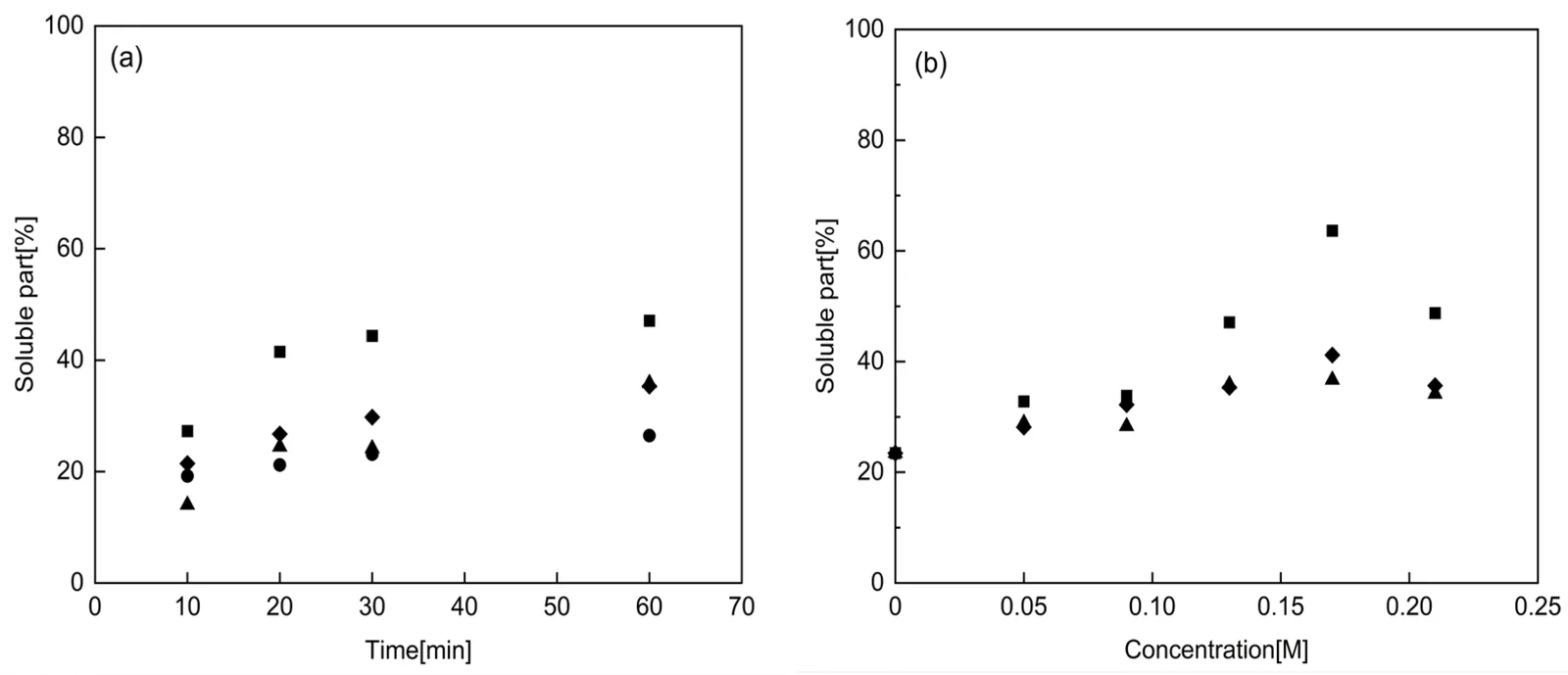
Soluble fraction of cellulose after pretreatment with (**a**) different milling times and (**b**) different additive concentrations. Cellulose: 4.375 g; glucose: 0.625 g; aqueous solution: 0.13 M, 10 mL for (**a**); ball milling: 60 min for (**b**). Notes: ■ [Bmim][OAc], ● [Bmim]Cl, ▲ H_2_SO_4_, and ◆ water.

The pretreatment conditions play an important role in determining the soluble fraction of cellulose. Therefore, the effects of milling time and additive concentration on cellulose solubilization were further investigated, as shown in Figure 3. As shown in Figure 3a, the soluble fraction generally increased with increasing milling time for all pretreatment conditions. The increase was more pronounced for [Bmim][OAc], whereas [Bmim]Cl, sulfuric acid, and water showed more gradual changes with milling time. This indicates that prolonged milling promoted cellulose solubilization, with [Bmim][OAc] showing the strongest response to milling time.

As shown in Figure 3b, the soluble fraction also varied with additive concentration. For [Bmim][OAc], the soluble fraction increased with increasing concentration up to 0.17 M, followed by a decrease at 0.21 M. Similar concentration-dependent trends were observed for [Bmim]Cl, sulfuric acid, and water, although the extent of the changes differed. The decrease at 0.21 M indicates that further increasing the additive concentration did not provide additional improvement in cellulose solubilization under the conditions investigated. The results demonstrated that both milling time and additive concentration affected cellulose solubilization, with [Bmim][OAc] showing the most pronounced response among the pretreatment conditions examined.

**Figure 4:**
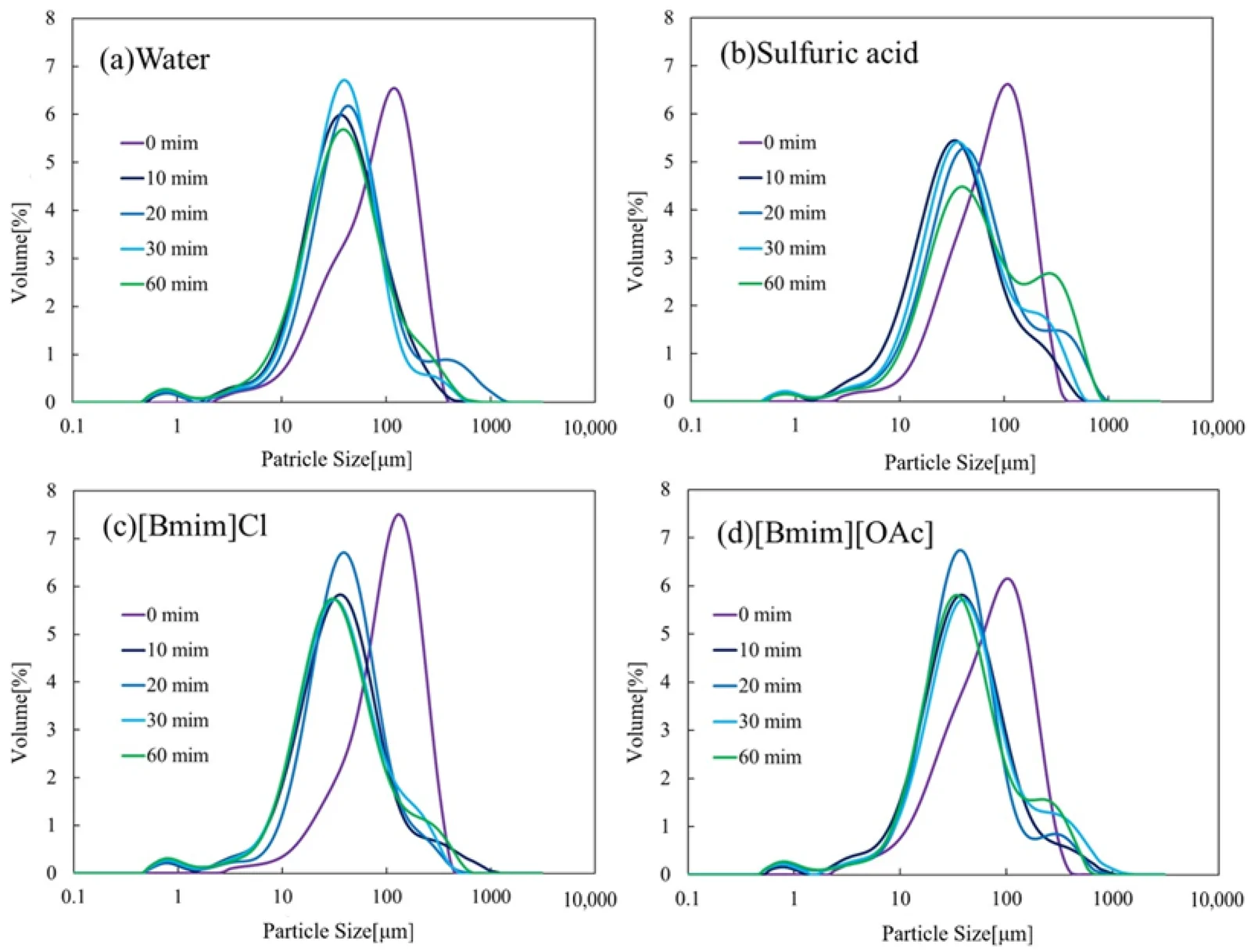
Time dependence of particle size distribution of pretreated cellulose with glucose addition. (Cellulose 4.375 g, Glucose 0.625 g, aqueous solution: 0.13 M 10 mL, ball milling: 0–60 min).

Figure 4 shows the effect of milling time on the particle-size distribution of cellulose pretreated with water, sulfuric acid, [Bmim]Cl, and [Bmim][OAc]. The particle-size distributions changed with increasing milling time, with the main peaks generally shifting toward smaller particle sizes compared with the unmilled samples. Particle-size characteristics at 20 min were further compared among the different pretreatment conditions, with the corresponding particle-size parameters summarized in Table 1. Among the pretreatment conditions, [Bmim]Cl exhibited the lowest number-average particle diameter of 54.04 µm, indicating the smallest average particle size among the treated samples. The unmilled cellulose exhibited its main particle-size peak at a larger particle-size region, whereas the milled samples showed their main peaks at smaller particle sizes, as shown in Figure S3.

**Figure 5:**
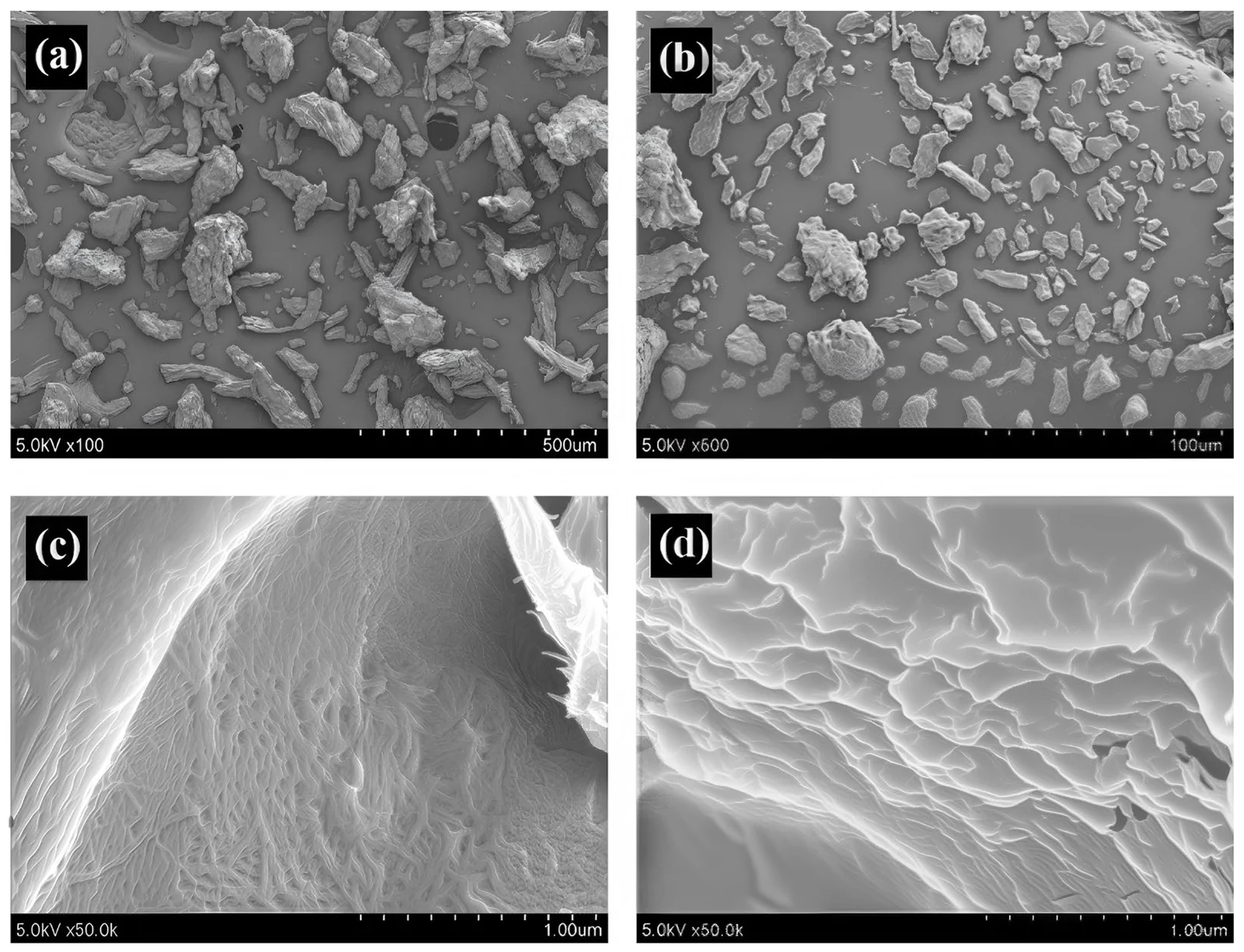
SEM images of pretreated cellulose with glucose addition: (**a**) aqueous solution without ball milling (magnification ×100); (**b**) [Bmim][OAc] aqueous solution with ball milling for 20 min (magnification ×600); (**c**) aqueous solution without ball milling (magnification ×50,000), and (**d**) [Bmim][OAc] aqueous solution with ball milling for 20 min (magnification ×50,000).

The morphology of the pretreated cellulose samples was examined by SEM (Figure 5). Figure 5a,b show the morphology of cellulose before and after ball milling treatment, respectively. At higher magnification, the untreated cellulose in Figure 5c exhibited a relatively smooth surface, whereas the ball-milled cellulose in Figure 5d showed a more irregular surface morphology. As stated by Zeng et al., ball milling generates mechanical forces, including impact, shear, compression, and friction [32], which can induce morphological changes in cellulose fibers. Therefore, the irregular surface features observed after ball milling may be attributed to structural modification of cellulose caused by these mechanical effects during treatment.

The effect of ball milling on cellulose crystallinity was further evaluated by XRD. As shown in Table 2, the crystallinity index decreased with increasing milling time for both ultrapure water and [Bmim][OAc] aqueous solution. The decrease in crystallinity indicates that ball milling disrupted the ordered structure of cellulose and increased the relative proportion of the amorphous structure. The reduction in crystallinity was more pronounced for the samples treated with [Bmim][OAc] aqueous solution at longer milling times, particularly at 30 and 60 min. This suggests that the presence of [Bmim][OAc] further promoted structural disruption during ball milling.

**Figure 6:**
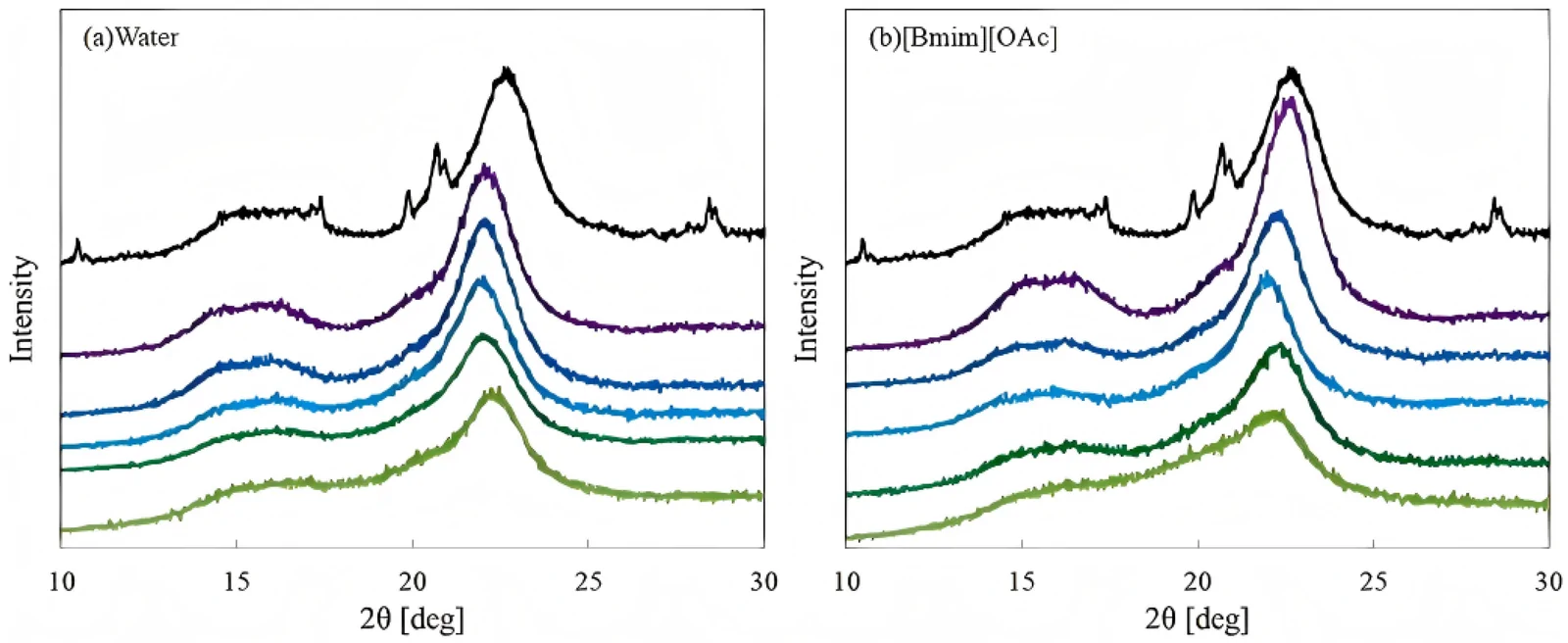
XRD patterns of pretreated cellulose with glucose addition. (Cellulose 4.375 g, Glucose 0.625 g, (**a**) ultrapure water (**b**) [Bmim][OAc] aqueous solution 0.13 M 10 mL). Notes: Black line: Raw Cellulose + Glucose, purple line: 0 min, dark blue line: 10 min, blue line: 20 min, bottle green line: 30 min, green line: 60 min.

The XRD patterns in Figure 6 show a decrease in crystallinity with increasing ball-milling time. As shown in Table 2, the crystallinity index (Cr_I_) decreased with increasing milling time for both water and [Bmim][OAc] aqueous solution. From 10 to 60 min, the Cr_I_ decreased by 14.47% and 34.19% for the water and [Bmim][OAc]-treated cellulose, respectively. This decrease can be attributed to the mechanical action of ball milling, which disrupts the highly ordered cellulose structure and promotes decrystallization [33]. Under wet milling conditions, prolonged milling can further enhance amorphization and reduce the crystallinity of cellulose [34]. Furthermore, Table 2 also reveals that the greater Cr_I_ decrease observed for [Bmim][OAc] indicates that the ionic liquid promoted greater disruption of the crystalline structure than water. This result corresponds with the higher soluble fraction obtained with [Bmim][OAc] compared with water (Figure 3a), suggesting that the lower crystallinity was associated with greater cellulose solubilization.

### 2.4. Characterization and Structural Reconstruction of Hydrotalcite Catalyst

**Figure 7:**
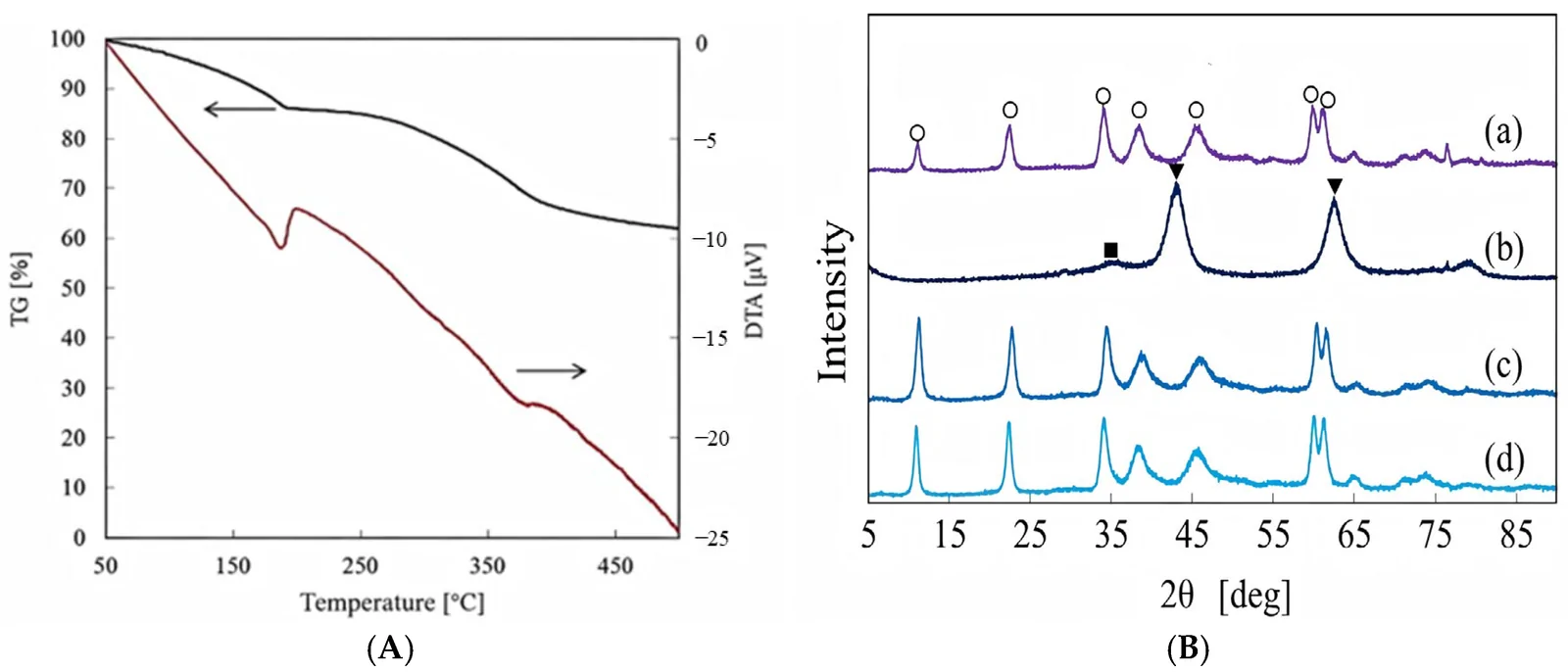
(**A**) TG-DTA curve of HT (hydrotalcite); (**B**) XRD patterns of the hydrotalcite samples: (a) HT, (b) HT500, (c) rehydrated HT, and (d) Ba(OH)_2_-rehydrated HT. Notes: ○: HT phase; ▼: MgO phase; ■: Al_2_O_3_ phase.

The TG-DTA profile of hydrotalcite (HT) in Figure 7A exhibited two main endothermic events accompanied by weight loss. The first event around 190 °C falls within the reported 50–300 °C range associated with the removal of interlayer water, while the second event near 370 °C falls within the 300–500 °C range associated with dehydroxylation, loss of interlayer carbonate species, and breakdown of the layered structure [35].

The structural changes during calcination and rehydration were examined by XRD (Figure 7B). The original HT sample (a) showed characteristic hydrotalcite peaks at approximately 11.6°, 23.3°, and 34.8°, corresponding to the (003), (006), and (009) planes, respectively, confirming the layered hydrotalcite structure. These peak positions agree well with those reported for Mg–Al hydrotalcites [36]. After calcination at 500 °C (b), the characteristic hydrotalcite peaks disappeared, while MgO-related peaks appeared together with a weak Al_2_O_3_-related peak, indicating the transformation of the layered HT structure into mixed oxide phases [37]. After rehydration, the characteristic HT peaks reappeared in both rehydrated HT (c) and Ba(OH)_2_-rehydrated HT (d), indicating reconstruction of the layered structure. This reconstruction is associated with the memory effect of Mg–Al hydrotalcites, whereby the calcined oxide can recover a hydrotalcite-like structure upon hydration [37].

### 2.5. Catalytic Conversion of Glucose to Lactic Acid

**Figure 8:**
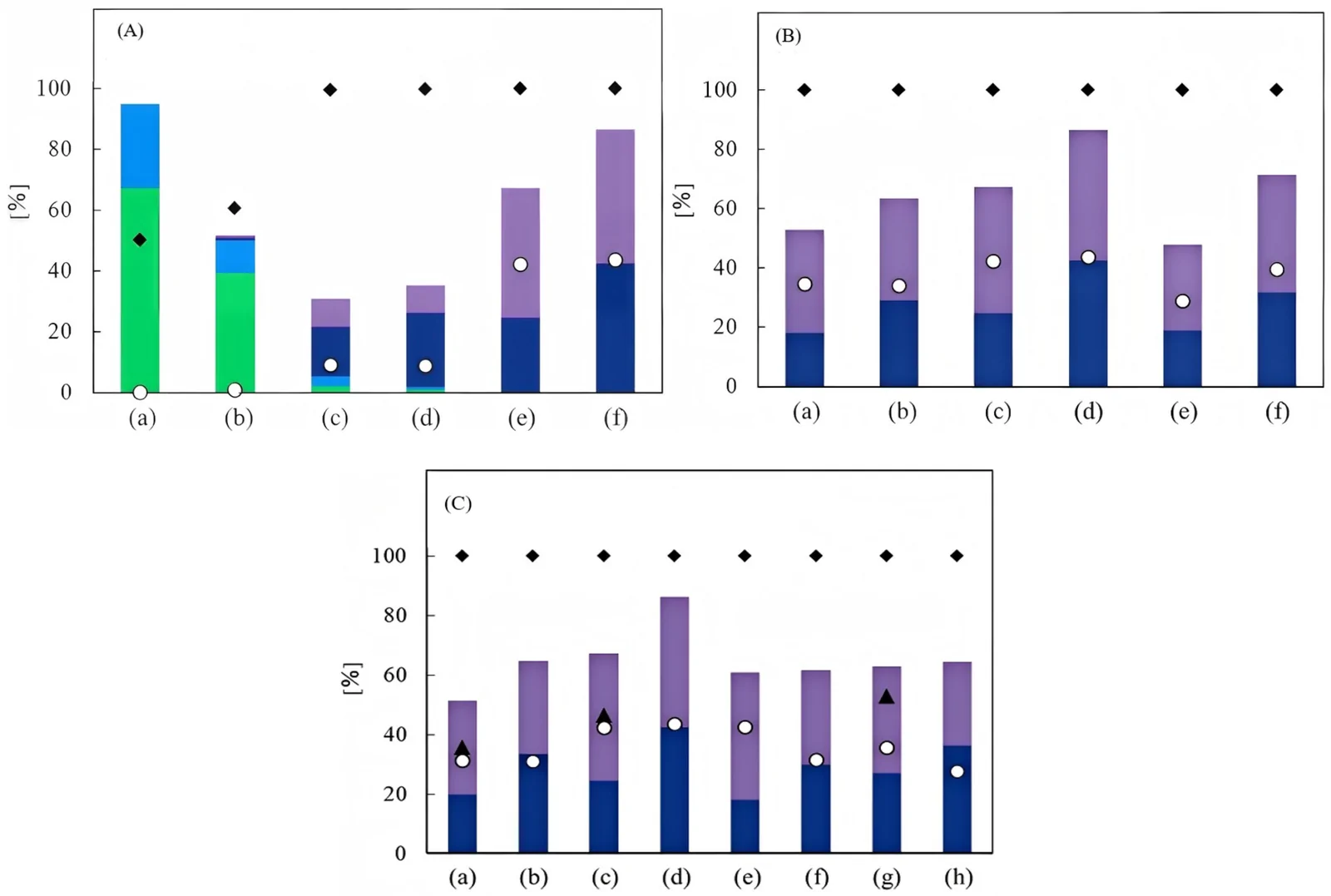
(**A**) Product yields, glucose conversion, and LA selectivity for different catalyst systems. (reaction conditions: 100 °C, 2 h, 0.25 M Ba(OH)_2_ solution: 2 mL, glucose: 0.036 g, catalyst: 0.1 g, (a)–(f): (a) no catalyst, water; (b) HT; (c) r-HT; (d) Ba-r-HT; (e) Ba(OH)_2_ solution; (f) r-HT + Ba(OH)_2_ solution.) and (**B**) effect of reaction temperature on product yields, glucose conversion, and LA selectivity (reaction conditions: 2 h, 0.25 M Ba(OH)_2_ solution: 2 mL, (a)–(f): (a) 80 °C without HT, (b) 80 °C with HT, (c) 100 °C without HT, (d) 100 °C with HT, (e) 120 °C without HT, (f) 120 °C with HT.) and (**C**) time course of product yields, glucose conversion, and LA selectivity (reaction conditions: 100 °C, 0.25 M Ba(OH)_2_ solution: 2 mL, (a)–(h): (a) 1 h without HT, (b) 1 h with HT, (c) 2 h without HT, (d) 2 h with HT, (e) 3 h without HT, (f) 3 h with HT, (g) 4 h without HT, (h) 4 h with HT). Notes: Green bar: Glucose, blue bar: Fructose, dark blue bar: Glyceraldehyde, purple bar: Lactic acid. Black diamond (◆): total conversion, white circle (○): yield, black triangle (▲): selectivity.

Figure 8A and Table 3 compare the glucose conversion and product yields for the different catalyst systems. In the absence of a catalyst, glucose conversion reached 50.1%, while the lactic acid yield was only 0.3 C-%. This indicates that water alone promoted glucose conversion to some extent but was insufficient to achieve substantial lactic acid formation. With the addition of uncalcined HT, glucose conversion increased to 60.6%, while the lactic acid yield remained low at 0.59 C-%, with glyceraldehyde and fructose yields of 0.74 and 7.8 C-%, respectively. These results indicate that uncalcined HT provided only a modest improvement in glucose conversion, while lactic acid formation remained limited.

In contrast, r-HT prepared by calcination at 500 °C followed by rehydration achieved complete glucose conversion, with lactic acid, glyceraldehyde, and fructose yields of 9.11, 16.1, and 3.3 C-%, respectively (Table 3). Despite complete glucose conversion, the relatively low lactic acid yield indicates that the converted glucose underwent further reactions rather than being converted predominantly to lactic acid. Under alkaline conditions, glucose can undergo isomerization to fructose, followed by retro-aldol reactions that generate smaller intermediates, which may undergo subsequent reactions toward lactic acid formation. The detection of glyceraldehyde at 16.1 C-% supports the formation of a C3 product in the reaction pathway. Such C3 intermediates are reactive and may undergo secondary reactions, which can limit lactic acid formation [38]. Furthermore, the higher glucose conversion with r-HT may be related to the reconstructed hydrotalcite structure, as evidenced by the reappearance of the characteristic hydrotalcite peaks in the XRD pattern after rehydration, indicating reconstruction of the layered structure (Figure 7B).

Although r-HT alone resulted in a relatively low lactic acid yield, adding r-HT to the Ba(OH)_2_ medium increased the lactic acid yield to 43.6 C-%, the highest among the catalyst systems (Table 3). Ba(OH)_2_ alone also showed a relative high lactic acid yield of 38.2 C-%, indicating its important contribution to glucose conversion toward lactic acid. This catalytic effect may be related to the contribution of both OH^−^ and Ba^2+^ during glucose conversion [39]. However, Ba(OH)_2_ alone can also promote side reactions during glucose isomerization and fructose retro-aldol reactions, which may limit lactic acid formation [7]. In the present study, introducing r-HT into the Ba(OH)_2_ medium increased the lactic acid yield from 38.2 to 43.6 C-% at 0.25 M glucose, giving the highest lactic acid yield among the catalyst systems investigated. This finding highlights the potential of r-HT as an effective solid-base component in Ba(OH)_2_-mediated glucose conversion and provides a promising approach for enhancing lactic acid production.

### 2.6. Effect of Reaction Temperature and Time

The effects of reaction temperature and time on lactic acid formation were investigated, with the temperature varied from 80 to 120 °C (Figure 8B). At 80 °C, the lactic acid yield was 18.7% without HT and slightly higher in the presence of HT, indicating limited lactic acid formation at the lower temperature. When the temperature increased to 100 °C, the lactic acid yield reached 43.6% for the r-HT and Ba(OH)_2_ system. However, further increasing the temperature to 120 °C decreased the lactic acid yield to approximately 35%. This may be due to further degradation of lactate to smaller organic acids under alkaline Ba(OH)_2_ conditions [40].

The effect of reaction time was then investigated at 100 °C for 1–4 h (Figure 8C). After 1 h, glucose conversion reached 100%, while fructose accumulated to 16.2% and the lactic acid yield was 12.5%. With increasing reaction time to 2 h, the fructose yield decreased while the lactic acid yield increased to 43.6%, indicating that the accumulated fructose was further converted toward lactic acid. This behavior can be explained by the sequential reaction pathway, in which glucose is first converted to fructose, followed by further conversion of fructose and C3 intermediates toward lactic acid [7]. When the reaction time was extended beyond 2 h, the lactic acid yield decreased to 38.2% at 3 h and 32.7% at 4 h. Since glucose conversion had already reached 100%, the decrease in lactic acid yield indicates that prolonged reaction favored further conversion or degradation of the formed products rather than additional glucose conversion.

## 3. Material and Methods

### 3.1. Materials

The ionic liquid 1-butyl-3-methylimidazolium bromide ([Bmim]Br, >97.0%), 1-butyl-3-methylimidazolium chloride ([Bmim]Cl, 98%), 1-butyl-3-methylimidazolium acetate([Bmim][OAc], 95.9%), 1-butyl-3-methylimidazolium hydrogen sulfate ([Bmim][HSO_4_], 95%), cellulose (microcrystalline, particle size 51 μm, bulk density 0.6 g/mL at 25 °C), D-(+)-Glyceraldehyde (≥98.0%) and Chloroform-d (99.8%) and deuterium oxide (99.8%) with tetramethylsilane (TMS, 0.03%) as an internal standard were purchased from Sigma Aldrich (Burlington, MA, USA). The glucose (98.0%), fructose (98%), lactic acid (85.0~92.0%), barium hydroxide anhydrous (≥98.0%), sulfuric acid (1 mol/L), sodium hydroxide (1 mol/L), dichloromethane (99.8%) and potassium hydroxide (85%) were purchased from Macklin (Shanghai, China).

### 3.2. Cellulose Pretreatment

The cellulose pretreatment experiments were conducted using different ionic liquids, including 1-butyl-3-methylimidazolium chloride ([Bmim]Cl), 1-butyl-3-methylimidazolium bromide ([Bmim]Br), 1-butyl-3-methylimidazolium hydroxide ([Bmim]OH), 1-butyl-3-methylimidazolium hydrogen sulfate ([Bmim][HSO_4_]), and 1-butyl-3-methylimidazolium acetate ([Bmim][OAc]). For the glucose-assisted experiments, glucose was added at quantities ranging from 0 to 0.625 g, corresponding to cellulose-to-glucose mass ratios of 7:1, while the respective ionic liquid and 98% sulfuric acid, when employed, were dissolved in 10 mL of ultrapure water. The resulting solution was added dropwise to cellulose (4.375–5.0 g) to obtain a total mass of 5 g. Both with-glucose and without-glucose conditions were investigated to evaluate the effect of glucose addition during pretreatment. Following impregnation, the samples were dried in a constant-temperature oven at 50 °C for 24 h to remove moisture, yielding dry microcrystalline cellulose (MCC) co-impregnated with the respective ionic liquid, with glucose and sulfuric acid included where applicable. Subsequently, 4.0 g of the dried MCC samples were subjected to ball milling using a mixer mill equipped with 5 mm diameter zirconia milling balls (Retsch GmbH, Haan, Germany) with a total mass of 64 g at a vibration frequency of 30 Hz. The ball-milling treatment was conducted for 10, 20, 30, and 60 min, with an untreated sample (0 min) used as the control. For the 60-min treatment, a 15-min cooling interval was introduced after the initial 30 min of milling to prevent overheating. Following milling, the pretreated cellulose samples were stored at −20 °C until further analysis.

### 3.3. Catalyst Preparation

Rehydrated hydrotalcite (r-HT) was obtained by calcination of HT followed by a rehydration treatment. HT was first placed in an electric tube furnace and purged with nitrogen (N_2_) at 0.1 MPa for 40 min. The sample was then heated to 500 °C at a constant heating rate of 5 K min^−1^ and maintained at this temperature for 6 h. After cooling to below 100 °C, the calcined product was collected and denoted as HT500. For rehydration, 2 g of HT500 was suspended in 200 mL of ultrapure water and stirred at 50 °C for 3 h. The resulting solid was separated by suction filtration and dried under vacuum at ambient temperature. The rehydrated product was stored in hermetically sealed containers to minimize exposure to atmospheric CO_2_.

Ba(OH)_2_-rehydrated HT was prepared using the same calcination procedure described above. Subsequently, 2 g of HT500 was suspended in 200 mL of saturated Ba(OH)_2_ solution, prepared by dissolving 15.8 g of Ba(OH)_2_·8H_2_O, and stirred at 50 °C for 3 h. After rehydration, the solid was collected by suction filtration, washed with ultrapure water, and dried under vacuum at ambient temperature. The resulting catalyst was stored in airtight containers to minimize exposure to atmospheric CO_2_ and preserve its structural characteristics.

### 3.4. Reaction Experiments

A stainless-steel batch reactor with an internal volume of approximately 5.7 mL was employed for the catalytic reactions. Prior to use, the reactor was passivated by charging with 3 g of 10.0% hydrogen peroxide solution and immersing in a fluidized sand bath at 400 °C for 30 min to form a protective oxide layer on the inner walls.

A 0.25 M barium hydroxide solution was prepared by dissolving 7.88 g of Ba(OH)_2_·8H_2_O in ultrapure water and diluting to a final volume of 100 mL. For each reaction, 0.036 g of D-glucose and 0.1 g of the designated HT catalyst were charged into the batch reactor. Subsequently, 2 mL of the Ba(OH)_2_ solution and a magnetic stirrer bar were added. The reactor was sealed and purged four times with nitrogen (N_2_), with the final pressure adjusted to 0.1 MPa. The reaction was conducted by immersing the reactor in an oil bath at temperatures ranging from 80 to 120 °C with agitation at 500 rpm for durations of 1 to 4 h.

Upon completion of the reaction, the mixture was recovered and the total mass was recorded. The mixture was subsequently transferred to a volumetric flask and diluted to 25 mL with ultrapure water. The solid residue was separated via suction filtration and weighed. For product analysis, 0.2 mL of 0.5 M H_2_SO_4_ was added to a 5 mL aliquot of the filtrate, resulting in the formation of BaSO_4_ precipitate and conversion of organic salts to free acids. Following mixing, 2 mL was centrifuged at 3000 rpm for 20 min to remove BaSO_4_. The supernatant was filtered through a 0.22 μm syringe filter for HPLC analysis.

### 3.5. Analytical Method

The soluble fraction of each pretreated cellulose sample was determined according to a procedure in which 0.36 g of sample was added to 7.2 mL of ultrapure water, followed by sonication in an ultrasonic bath for 15 min and vortex mixing for 3 min at an intensity setting of 7 to ensure adequate dispersion. The resulting mixture was then vacuum-filtered through a 0.20 μm membrane filter. The soluble fraction ratio was calculated according to Equation (1):

(1) $$S=\frac{\left(W_{\left\{total\right\}}-W_{\left\{residual\right\}}\right)}{W_{\left\{total\right\}}}$$

where S represents the soluble fraction ratio [−], $W_{\left\{total\right\}}$ denotes the total mass of cellulose [g], and $W_{\left\{residual\right\}}$ denotes the mass of the residual cellulose [g].

Fourier-transform infrared spectra of cellulose samples before and after milling were recorded on a Fourier-transform infrared spectrophotometer in the wavenumber range of 650–4000 cm^−1^, and the assignment of each absorption band was performed. Potassium bromide and the test sample were pulverized in an agate mortar to obtain a fine powder. The mixture was thoroughly blended so that potassium bromide constituted approximately 1% relative to the test sample. Background measurement was performed using potassium bromide as the reference sample, followed by sample measurement.

The thermal stability and weight loss behavior of cellulose samples before and after milling were investigated using a simultaneous TG/DTA analyzer (Shimadzu DTG60A, Kyoto, Japan). For each measurement, less than 10 mg of sample was placed in an aluminum pan. The analysis was conducted under a nitrogen (N_2_) atmosphere over a temperature range of 25–500 °C, employing a heating rate of 5 °C min^−1^ and a cooling rate of 100 °C min^−1^, followed by an isothermal hold at ambient temperature for 20 min.

The particle size distributions of cellulose samples before and after milling were measured using a laser diffraction particle size analyzer (Malver Mastersizer S3000, Malvern Instruments Ltd., Malvern, UK). The number-average particle size, mass-average particle size, and Span were defined according to Equations (2)–(4), respectively. The number-average particle size represents the mean diameter based on particle count, whereas the mass-average particle size is based on particle mass. The Span serves as an indicator of the width of the size distribution, with larger values signifying broader distributions.

(2) $$\bar{{\left\{d\right\}}_{N}}\left[\mu m\right]=\frac{\sum n_{i}d_{i}}{\sum n_{i}}$$

(3) $$\bar{{\left\{d\right\}}_{w}}\left[\mu m\right]=\frac{\sum n_{i}m_{i}d_{i}}{\sum n_{i}m_{i}}=\frac{\sum n_{i}d_{i}^{4}}{\sum n_{i}d_{i}^{3}}$$

(4) $$Span\left[-\right]=\frac{\left(d_{90}-d_{10}\right)}{d_{50}}$$

where $\bar{{\left\{d\right\}}_{N}}\left[\mu m\right]$ and $\bar{{\left\{d\right\}}_{w}}\left[\mu m\right]$ represent the number-average and mass-average particle sizes, respectively; $n_{i}$[%] is the percentage frequency of particles with diameter $d_{i}\left[\mu m\right]$; and $m_{i}\left[g\right]$ denotes the mass of particles with diameter $d_{i}\left[\mu m\right]$. Furthermore, $d_{10}$, $d_{50}$, and $d_{90}\left[\mu m\right]$ are the particle diameters corresponding to 10%, 50%, and 90% of the cumulative frequency distribution.

The surface morphologies of cellulose samples before and after milling were examined using a field-emission scanning electron microscope (FESEM) (Tescan CLARA, Tescan Orsay Holding a.s., Brno, Czech Republic). For sample preparation, a small quantity of each cellulose sample was deposited onto carbon-coated film grids. Observation was conducted at an accelerating voltage suitable for high-resolution imaging.

The crystallinity index (Cr_I_) of various pretreated cellulose samples was determined using an X-ray diffractometer (D8 Advance instrument, Bruker, Germany). Powder samples were packed into aluminum pans and leveled flush with the surface to ensure random orientation. XRD patterns were recorded over a 2θ range of 5° to 30°. The X-ray generator was operated at 30 kV and 15 mA, with a scattering slit of 4.2° and a receiving slit of 0.3 mm. The crystallinity index, representing the relative proportion of crystalline and amorphous structures within the microcrystalline cellulose samples, was calculated according to Equation (5):

(5) $$Cr_{I}\left[-\right]=\frac{\left(I_{002}-I_{am}\right)}{I_{002}}\times 100$$

where $Cr_{I}\left[-\right]$ represents the relative crystallinity index; $I_{002}$ is the maximum intensity of the diffraction from the (002) lattice plane at 2θ = 22.8°; and $I_{am}$ is the diffraction intensity from the amorphous region at 2θ = 18°.

The concentrations of glucose, fructose, glyceraldehyde, and lactic acid in the reaction products were quantified using an HPLC system equipped with a guard column and analytical columns in series. The mobile phase was 5 mmol L^−1^ H_2_SO_4_ at a flow rate of 0.5 mL min^−1^. Detection was performed using a refractive index detector and a photodiode array detector at 210 nm. The column temperature was maintained at 50 °C. Calibration curves were prepared using standard reagents for quantitative analysis. Glucose conversion and carbon-based product yields were calculated using Equations (6) and (7).

(6) $$\mathrm{Substrate}\;\mathrm{conversion}\;\left[\%\right]=\left\{1-\left(\frac{\mathrm{Residual}\;\mathrm{glucose}\;\mathrm{molar}\;\mathrm{concentration}\;\left[M\right]}{\mathrm{Initial}\;\mathrm{glucose}\;\mathrm{molar}\;\mathrm{concentration}\;\left[M\right]}\right)\right\}\times 100\;$$

(7) $$\begin{array}{c} Product\;yield\;\left[\%\right]=\left(\begin{array}{c} \frac{\mathrm{Product}\;\mathrm{molar}\;\mathrm{concentration}\;\left[M\right]}{\mathrm{Initial}\;\mathrm{glucose}\;\mathrm{molar}\;\mathrm{concentration}\;\left[M\right]}\\ \times \frac{\mathrm{Number}\;\mathrm{of}\;\mathrm{carbons}\;\mathrm{per}\;\mathrm{mole}\;\mathrm{of}\;\mathrm{product}\;\left[-\right]}{\mathrm{Number}\;\mathrm{of}\;\mathrm{carbons}\;\mathrm{per}\;\mathrm{mole}\;\mathrm{of}\;\mathrm{substrate}\;\left[-\right]} \end{array}\right)\times 100 \end{array}$$

The carbon yield of each identified product was calculated by considering the number of carbon atoms in the product relative to glucose. The reaction resulted in both identified and unidentified products. Following the approach adapted from Onda et al., the unidentified products were denoted as “others” [39]. The carbon yield of “others” was estimated by subtracting the carbon yields of the remaining glucose and identified products from the initial carbon introduced, as follows:

(8) $$C-\%\;Others\;=100-\left(C-\%\;glucose\;remaining+C-\%\;fructose+C-\%\;glyceraldehyde+C-\%\;LA\right)$$

where the “others” fraction represents unidentified products that were not quantitatively determined by the present HPLC analysis.

## 4. Conclusions

In conclusion, this study demonstrates a two-stage approach combining mechanochemical ionic-liquid pretreatment of cellulose with subsequent catalytic conversion of glucose to lactic acid. Ball milling with [Bmim][OAc] increased the soluble fraction from 5.1% to 63.7% after 60 min, while the crystallinity index decreased from 78.1% to 44.0%, demonstrating effective structural modification of cellulose. In the catalytic stage, uncalcined HT provided only a modest improvement in glucose conversion, whereas r-HT achieved complete glucose conversion after calcination and rehydration. Although r-HT alone produced only 9.1 C-% lactic acid, the addition of r-HT to the Ba(OH)_2_ medium increased the yield to 43.6 C-%, the highest among the catalyst systems investigated, compared with 38.2 C-% for Ba(OH)_2_ alone. The reaction-condition study further showed that lactic acid formation was favored at 100 °C and 2 h, while higher temperature or longer reaction time resulted in lower yields. Overall, these results demonstrate that mechanochemical pretreatment improves cellulose accessibility, while the r-HT/Ba(OH)_2_ system enhances lactic acid formation, highlighting its potential for the valorization of cellulose-derived carbohydrates into value-added lactic acid.

## Figures and Tables

**Table 1:** Number-average particle diameter, weight-average particle diameter, and Span of pretreated cellulose with glucose addition. (Cellulose 4.375 g, Glucose 0.625 g, aqueous solution: 0.13 M 10 mL, ball milling: 20 min).

Solvent	Span [−]	$\bar{d_{N}}$ (μm)	$\bar{d_{w}}$ (μm)
No milling	2.198	98.22	215.6
Water	3.755	88.63	794.5
Sulfuric acid	5.694	95.69	551.1
[Bmim]Cl	2.460	54.04	279.1
[Bmim]OH	2.795	68.13	605.3
[Bmim][HSO_4_]	3.755	62.38	387.2
[Bmim]Br	3.755	64.40	393.4
[Bmim][OAc]	2.795	61.47	467.3

**Table 2:** Time dependence of the crystallinity index of pretreated cellulose with glucose addition. (Cellulose: 4.375 g; glucose: 0.625 g; aqueous solution: 0.13 M, 10 mL).

Solvent	Time [min]	I_am_ [a.u.]	I_002_ [a.u.]	Cr_I_ [−]
none	0	775	3533	78.06
Water	0	676	3370	79.94
	10	811	3505	76.86
	20	768	3028	74.64
	30	671	2175	69.15
	60	1011	2688	62.39
[Bmim][OAc]	0	911	4436	79.89
	10	680	3123	78.23
	20	760	2795	72.81
	30	951	2800	66.04
	60	1408	2516	44.04

**Table 3:** Catalytic conversion of glucose and distribution of carbon-based products over different catalyst systems (100 °C, 2 h, 0.25 M Ba(OH)_2_).

Catalyst	Conversion [%]	LA Yield [C-%]	Glyceraldehyde Yield [C-%]	Fructose Yield [C-%]	Others [C-%]
No catalyst, water	50.1	0.3	0	5.2	44.6
HT	60.6	0.59	0.74	7.8	51.47
r-HT	100	9.11	16.1	3.3	71.49
Ba-r-HT	100	12.3	18.7	0.96	68.04
Ba(OH)_2_ solution	100	38.2	28.9	0	32.9
r-HT + Ba(OH)_2_	100	43.6	48	0	8.4

## Data Availability

All relevant data are within the manuscript.

## Supplementary Materials

The following supporting information can be downloaded at https://www.mdpi.com/article/10.3390/molecules31162922/s1, Figure S1. (a) ^1^H-NMR analysis results of [Bmim]OH (b) Structural formula of [Bmim]OH; Figure S2. FT-IR analysis results of [Bmim]OH; Figure S3. Particle size distribution of pretreated cellulose with glucose addition. (cellulose 4.375 g, glucose 0.625 g, aqueous solution: 0.13 M 10 mL, ball milling: 20 min); Figure S4. Hydrotalcite (HT) structural transformation reaction of Mg-Al oxide; Table S1. Identification results of ^1^H-NMR analysis of [Bmim]OH; Table S2. Identification results of FT-IR analysis of [Bmim]OH.
