# Supplementary material for: An Integrated Two-Stage Strategy for Efficient Lactic Acid Production via Ionic Liquid-Assisted Mechanochemical Pretreatment and Catalytic Conversion
Source: Molecules. 2026 Aug 21;31(16):2922. doi: 10.3390/molecules31162922 (PMC13515166; doi:10.3390/molecules31162922)
Supplement: Supplementary file 1 [file molecules-31-02922-s001.zip › molecules-4462306-supplementary.pdf]

*Supplementary Materials*

# An Integrated Two-Stage Strategy for Efficient Lactic Acid Production via Ionic Liquid-Assisted Mechanochemical Pretreatment and Catalytic Conversion

**Zheyuan Zhang [1,2], Chuan Li Lee [2,3,*], Jianrong Shan [2] and Haixin Guo [2,*]**

[1] College of Resource and Environment, Hubei University, 368 Youyi Avenue, Wuchang District, Wuhan 430062, China
[2] Agro-Environmental Protection Institute, Ministry of Agriculture and Rural Affairs, No. 31 Fukang Road, Nankai District, Tianjin 300191, China; 82101221187@caas.cn
[3] Faculty of Biotechnology and Biomolecular Sciences, Universiti Putra Malaysia, Serdang 43400, Selangor, Malaysia
* Correspondence: chuanli@upm.edu.my (C.L.L); guohaixin@caas.cn (H.G)

## Section S1. Preparation of [Bmim]OH

1-Butyl-3-methylimidazolium hydroxide ([Bmim]OH) was prepared according to a literature procedure. In a 50 mL round-bottom flask, 2.3 g of KOH (40 mmol), 8.8 g of [Bmim]Br (40 mmol), and 20 mL of dichloromethane were added and stirred at 500 rpm at room temperature. After 10 h, the resulting solution was separated by filtration through a 0.22 μm membrane filter and evaporated at 60 °C under vacuum. The vacuum-dried liquid was subsequently washed twice with 20 mL of ethyl acetate per wash and dried at 60 °C under vacuum. The resulting liquid was further analyzed by NMR and FT-IR.

### $^1$H-NMR

The $^1$H-NMR spectrum of the synthesized [Bmim]OH was recorded on a spectrometer operating at 400 MHz and equipped with a 5 mm BBFO probe. The chemical shifts were observed and peak assignments were performed accordingly. For the measurement, the sample was dissolved in 0.8 mL of $CDCl_3$ containing 0.03% TMS at a concentration of several volume percent. The analysis was conducted in a standard NMR tube at 25 °C with a total of 32 scans.

In addition, the mixture was stirred at room temperature in a 50 mL Erlenmeyer flask for 10 h, and KBr precipitation was subsequently confirmed. Furthermore, Fig. S1 confirms that the [Bmim] structural framework was preserved after the reaction.

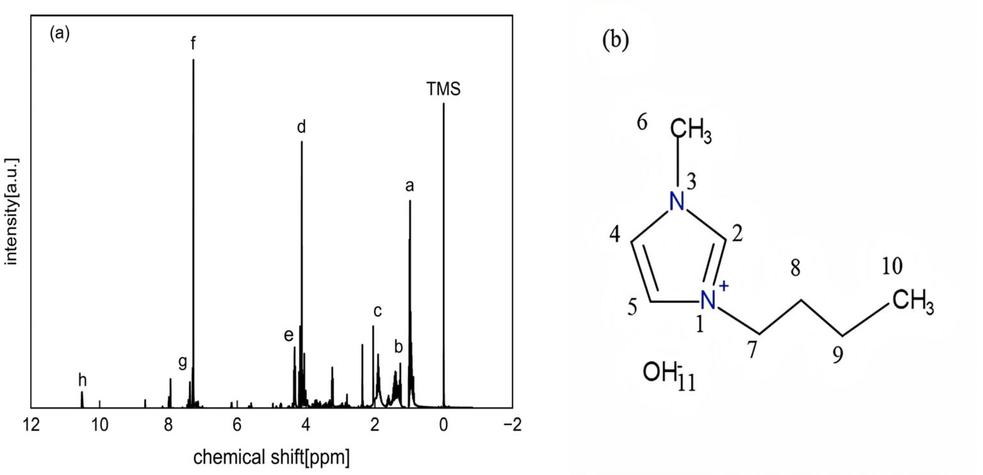


**Figure S1.** (a) $^1$H-NMR analysis results of [Bmim]OH (b) Structural formula of [Bmim]OH.

**Table S1.** Identification results of $^{1}$H-NMR analysis of [Bmim]OH.

| Entry | Chemical shift [ppm] | Multiplicity | integration ratio | identification results |
|---|---|---|---|---|
| a | 0.83 | Triplet | 3 | $C_{(10)}$-$H_3$ |
| b | 1.15-1.32 | Multiple | 2 | $C_{(8)}$-$H_2$ |
| c | 1.76-1.86 | Multiple | 2 | $C_{(9)}$-$H_2$ |
| d | 4.00 | Singlet | 3 | $C_{(6)}$-$H_3$ |
| e | 4.23 | Triplet | 2 | $C_{(7)}$-$H_2$ |
| f | 7.46 | Doublet | 1 | $C_{(4,5)}$-H |
| g | 7.59 | Doublet | 1 | $C_{(4,5)}$-H |
| h | 10.15 | Singlet | 1 | $C_{(2)}$-H |

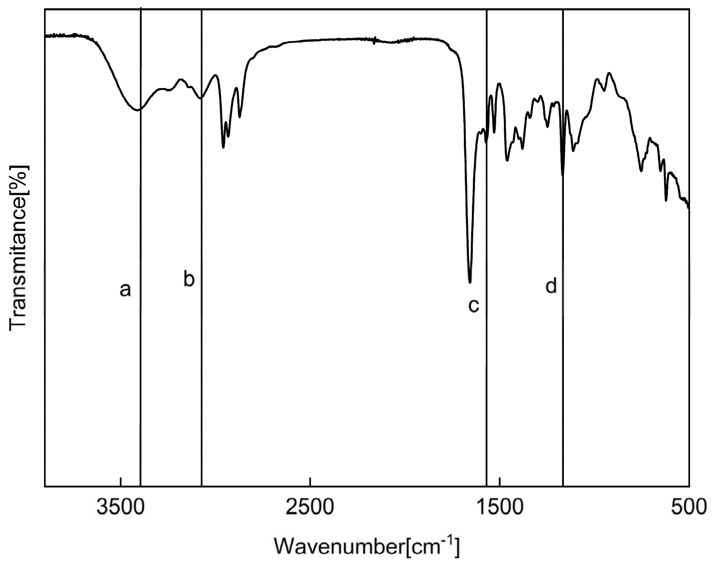


**Figure S2.** FT-IR analysis results of [Bmim]OH.

**Table S2.** Identification results of FT-IR analysis of [Bmim]OH.

| Entry | Wavelength [$cm^{-1}$] | Functional group identification |
|---|---|---|
| a | 3435 | O-H Telescopic |
| b | 3060 | N-$CH_3$ Telescopic |
| c | 1569 | Imidazolium Ring Vibration |
| d | 1168 | C-C-N Telescopic |

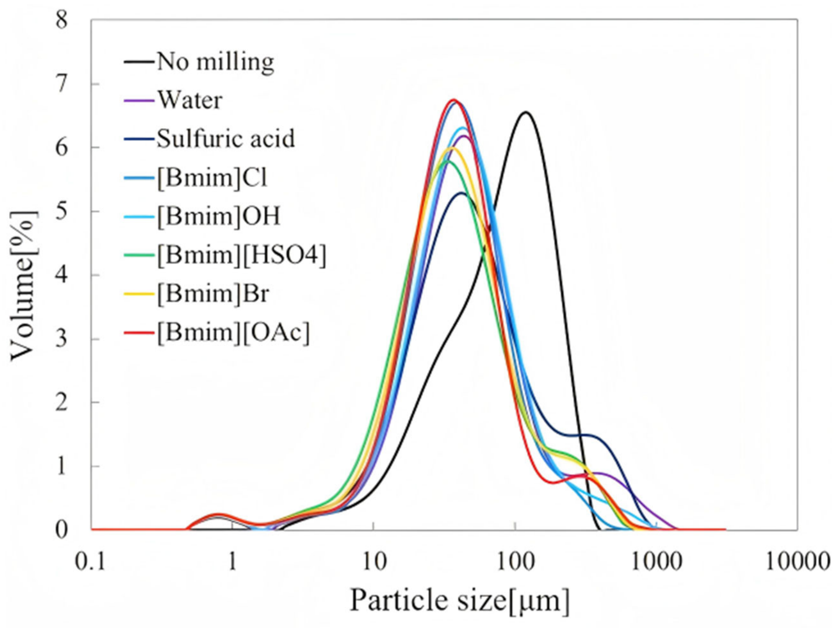


**Figure S3.** Particle size distribution of pretreated cellulose with glucose addition. (cellulose 4.375 g, glucose 0.625 g, aqueous solution: 0.13 M 10 mL, ball milling: 20 min).

## Section S2 Catalyst characterization

Figure S4 illustrates the structural transformation reactions of Mg-Al oxide to HT.

$$Mg_6Al_2(OH)_{16}CO_3 \rightarrow Mg_6Al_2O_9 + CO_2 + 8H_2O \quad (S1)$$

$$Mg_6Al_2O_9 + 9H_2O \rightarrow Mg_6Al_2(OH)_{18} \quad (S2)$$

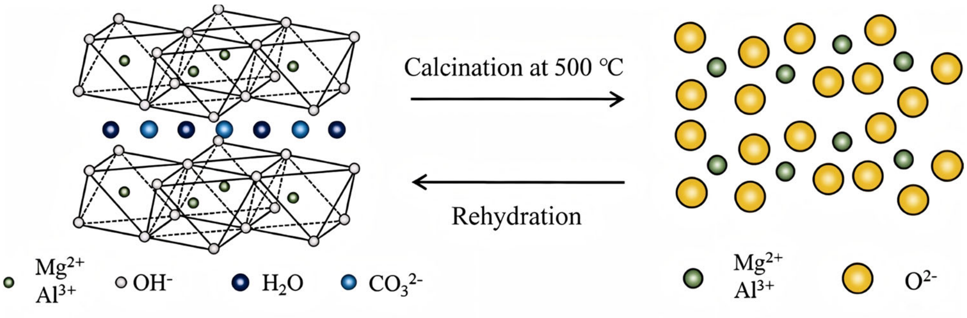


**Figure S4.** Hydrotalcite (HT) structural transformation reaction of Mg-Al oxide.
